# Multimorbidity is associated with the income, education, employment and health domains of area-level deprivation in adult residents in the UK

**DOI:** 10.1038/s41598-022-11310-9

**Published:** 2022-05-04

**Authors:** Gundi Knies, Meena Kumari

**Affiliations:** 1 Institute of Rural Studies, Johann Heinrich Von Thünen-Institut, Bundesallee 64, 38116 Braunschweig, Germany; 2 Institute for Social and Economic Research (ISER), University of Essex, Wivenhoe Park, Colchester, CO4 3SQ UK

**Keywords:** Epidemiology, Socioeconomic scenarios

## Abstract

Evidence suggests that there are social inequalities in multimorbidity, with a recent review indicating that area levels of deprivation are consistently associated with greater levels of multimorbidity. Definitions of multimorbidity, the most common of which is the co-occurrence of more than one long term condition, can include long term physical conditions, mental health conditions or both. The most commonly used measure of deprivation in England and Wales is the Index of Multiple Deprivation (IMD), an index of seven different deprivation domains. It is unclear which features of IMD may be mediating associations with multimorbidity. Thus, there may be associations because of the individual characteristics of those living in deprived areas, characteristics of the areas themselves or overlap in definitions. Data from over 25,000 participants (aged 16+) of *Understanding Society* (Wave 10, 1/2018–3/2020) were used to understand the most salient features of multimorbidity associated with IMD and whether physical or mental conditions are differentially associated with the seven domains of IMD. 24% of participants report multimorbidity. There is an increased prevalence of multimorbidity composed of only long-term physical conditions in the most deprived decile of deprivation (22%, 95% CI[19,25]) compared to the least deprived decile (16%, 95% CI[14,18]). Mental health symptoms but not reporting of conditions vary by decile of IMD. Associations with multimorbidity are limited to the health, income, education and employment domains of IMD. We conclude that multimorbidity represents a substantial population burden, particularly in the most deprived areas in England and Wales.

## Introduction

Social inequalities in mortality persist in high-income countries with universal health care, but the mechanisms by which these inequalities are generated remain unclear. The importance of multimorbidity is increasingly being recognised^1^, particularly at older ages^2^, but it is not uncommon for an individual to experience multimorbidity before old age^3^. About 15–25% of children and adolescents have a chronic physical health condition^4,5^, and there is a strong link between chronic physical illness and mental health problems, including among adolescents^6^. There is also a rapid rise in the prevalence of poor mental health among young people, rates in England having increased from 11.4% in 1999 to 15.3% in 2017; in 2017, 6.3% of young people met the criteria for two or more mental disorders^7^. It is, therefore, likely that multimorbidity is on the rise in all age groups.

Evidence suggests that there are social inequalities in multimorbidity2. A recent review of the literature of the association of household and area-level social determinants of health suggested that the most robust and consistent associations were found for area-level deprivation, measured using the Index of Multiple Deprivation (IMD), the official measure of relative deprivation at small scales in England and Wales^8^. It is unclear which features of IMD may be mediating these associations. Thus, there may be associations because of the individual characteristics of those living in deprived areas, characteristics of the areas themselves or overlap in definitions. A recent report examining the role of health behaviours in the association of area-level socioeconomic disadvantage suggested that these lifestyle factors only partially mediated these association^9^.

Better monitoring of multimorbidity and timely interventions might help to improve population health. However, there are several gaps in the literature.

Firstly, the definition of multimorbidity is unclear, and researchers have adopted many ways of defining multimorbidity. Thus, multimorbidity has been created as a simple score of the number of conditions. Previously this count has varied from 3 to 130 and is often limited by data availability. The most common way to define multimorbidity is the presence of more than one chronic condition^10^.

More recently, mental and physical multimorbidity has been separated in analyses and reveals different patterns by age with deprivation^11^. While an association of multimorbidity by IMD might be expected, it is unclear whether the physical or mental condition components of multimorbidity drive these associations.

Secondly, it is unclear whether multimorbidity reflects the health domain of IMD. Using the 2004 IMD, previous research suggested that studies of health inequalities should not include the health domain in the IMD^12^ due to the potential of ‘mathematical coupling’^13^, “a phenomenon whereby two variables will inevitably correlate if one contains or shares, directly or indirectly, all or part of the other”^14^. Specifically, the health domain of IMD uses a count of the number of people suffering from anxiety disorders and an estimate of the number of years of life expectancy lost due to ill health^15^. The income and employment domains of IMD, too, may be closely linked to multimorbidity because individuals with severe health problems have a lower labour market attachment and are entitled to social security payments^16^, which are the characteristics captured in these domains of area-level deprivation. With mental health issues on the rise, the extent of mathematical coupling warrants a re-examination.

We aim to use data available in *Understanding Society* to address the following aims:

- To describe the association of multimorbidity with IMD
- To understand which are the most salient features of multimorbidity associated with IMD; physical or mental conditions
- To examine whether multimorbidity, physical or mental conditions are differentially associated with the seven domains of IMD.

## Methods

### Study sample and design

Data are from *Understanding Society* (UKHLS)^17^, a longitudinal, nationally representative study of UK households. The study was established in 2009 and all adult household members are interviewed annually. Interviews are computer-assisted and take place over a 24 months fieldwork period. We use data from the latest (tenth) round of annual interviews in which participants were interviewed in person in their home or on-line. For more detailed information about the *Understanding Society* design, see the study user guide^18^. All participants in our survey gave oral consent at each wave of data collection. Participants were enrolled only after informed consent was provided. Overall, 28,523 individuals aged 16 years or older living in England and Wales participated. The University of Essex Ethics Committee has approved all data collection on *Understanding Society* main study. All methods were carried out in accordance with the approved guidelines and regulations.

### Variables and measurement

Our key outcome variables are based on the respondents’ self-reports of whether a medical doctor had ever told them they had any of a series of listed chronic conditions, see Table 1.

**Table 1:** List of chronic health conditions surveyed in *Understanding Society.*

Chronic health conditions			Group
Physical conditions	1	Asthma	Respiratory
	2	Emphysema	
	3	Chronic bronchitis	
	4	Chronic Obstructive Pulmonary Disease (COPD)	
	5	Congestive heart failure	Cardiovascular
	6	Coronary heart disease	
	7	Angina	
	8	Heart attack or myocardial infarctions	
	9	Stroke	
	10	Bowel/colorectal cancer	Cancers
	11	Lung cancer	
	12	Breast cancer (females only)	
	13	Prostate cancer (males only)	
	14	Liver cancer	
	15	Skin cancer or melanoma	
	16	Other cancer	
	17	High blood pressure/hypertension	Obesity related
	18	Type 2 diabetes	
	19	Gestational diabetes (females during pregnancy)	
	20	Other diabetes	
	21	Osteoarthritis	Arthritis
	22	Rheumatoid arthritis	
	23	Other arthritis	
	24	Hypothyroidism or an under-active thyroid	Autoimmunity
	25	Type 1 diabetes	
	26	Any kind of liver condition	Other chronic condition
	27	Multiple Sclerosis	
	28	HIV	
	29	Other longstanding/chronic condition	
Mental conditions	30	Epilepsy	Psychiatric conditions
	31	Psychosis or schizophrenia	
	32	Bipolar disorder or manic depression	
	33	Anxiety	Other mental health conditions
	34	Depression	
	35	An eating disorder	
	36	Post-traumatic stress disorder (PTSD)	
	37	Other emotional, nervous or psychiatric problem	

We computed various morbidity measures. Firstly, a simple count of the total number of conditions a participant has been diagnosed with ranging from a possible 0–36 for women (men: 0–35), and separate scores for physical conditions (max score women: 28, men: 27) and mental conditions (max score: 8). Next, we created three multimorbidity indicator variables. The first multimorbidity indicator considers multimorbid those with two or more physical conditions; the second considers those with any two or more health conditions (i.e., physical or mental conditions). As some of the listed conditions tend to co-occur or are known under different names by different cohorts of people, we also grouped conditions into nine broader groups (see Table 1). Therefore, the maximum possible score is nine, and we consider participants with scores of two or above multimorbid.

To explore the potential under-reporting of mental conditions in a face-to-face interview and non-diagnosing of mental health issues, particularly in more deprived areas, we draw on the GHQ-12, which is collected as part of a questionnaire completed by the participant. The GHQ-12 is designed to capture depressive and anxiety symptoms and a widely used non-psychotic psychological distress measure with excellent psychometric properties^19^. Each item has four response categories on a Likert scale ranging from ‘not at all’ to ‘much more than usual’. Those responding to an item as 'rather more' or 'much more than usual' are scored as^1^ and those responding as 'not at all' or 'no more than usual' are scored as [0]; negatively worded items are reverse-coded. Scores are summed and range from 0 to 12. Psychological distress 'caseness' is defined as a score of 4 or above^20^.

The main covariate of interest is the 2019 Index of Multiple Deprivation (IMD)^15^. The IMD is a measure of relative area disadvantage along seven weighted domains of deprivation (2019 weights in italics): Income deprivation (*22.5%*), Employment deprivation (*22.5%*), Education, skills and training deprivation (*13.5%),* Health deprivation and Disability (*13.5%*), Crime (*9.3%*), Barriers to housing and services (*9.3%*), and Living environment deprivation (*9.3%*). We use deciles of the 2019 IMD and its seven constituent indicators. The lower the decile, the higher the relative (domain) deprivation in the neighbourhood.

Area deprivation is not observed directly in the study but available at the spatial scale of 2011 Lower Super Output Areas (LSOA) through geographical data linkage drawing on the postcode of survey participants’ addresses and the Office for National Statistics Postcode Directory (ONSPD)^21^. LSOA are official spatial reporting units designed to refer to localities that local people conceive as neighbourhoods; they are socio-economically homogeneous and have an average population of 600 households (~ 1500 people).

We also consider the participants’ living environment beyond its relative deprivation level using the Office for National Statistics (ONS)’ Classification of Workplace Zones (COWZ) 2011. There are 60,709 Workplace Zones (WZs) in the UK, and the classification describes the characteristics of the working population in those areas based on a cluster analysis that draws on 48 characteristics collected in the 2011 census^22^. Like the IMD, this classification is available through geographical data linkage using the ONSPD.

In addition, a broad range of social and demographic factors are collected in the *Understanding Society* study, and we use the following: Age, sex (women and men), ethnicity (White British, Caribbean/African Black, Indian, Pakistani, Bangladeshi, Other Asian, Other), social class of the main job (NS-SEC 8, but with the not currently employed population differentiated into the categories ‘retired’, ‘longstanding health condition or disability and ‘other’ based on the main economic activity status).

All participants with complete information on chronic health conditions, socio-demographics and neighbourhood characteristics are included in the analysis (N = 24,520). For detailed variable and sample descriptions, see Supplementary Table S1 online. In the statistical analysis, we use the individual response weights for 2018/19 provided with the study data and adjust standard errors for clustering and stratification to account for unequal selection and response probabilities.

## Results

### Prevalence of multimorbidity and association with neighbourhood deprivation

Multimorbidity is a phenomenon that affects 24% of the population aged 16 years or older living in England and Wales; 76% of the population have been diagnosed with either none or one condition (Table 2). 18% of the population (representing 75% of the multimorbid population) have multiple physical conditions only, 1% have multiple mental conditions only, and 5% have a mixed conditions-profile. On average, the population has less than one of the 35 listed health conditions, with the average number of physical conditions exceeding the average number of mental conditions (0.84 compared to 0.13, respectively).

**Table 2:** Multimorbidity in England and Wales 2018/19.

	Mean	95% Confidence Interval	
Number of chronic health conditions	0.98	0.96	1.01
**… By type of condition:**			
Physical conditions	0.85	0.83	0.87
Mental conditions	0.13	0.12	0.14
No multimorbidity (0 or 1 conditions)	0.76	0.75	0.76
Multimorbidity (> 1 condition)	0.24	0.24	0.25
**… By physical/mental conditions mix**			
Only physical conditions	0.18	0.18	0.19
Only mental conditions	0.01	0.01	0.02
Mixed	0.05	0.04	0.05
**Multimorbidity by type of conditions**^**1**^			
No chronic conditions	0.50	0.49	0.51
One type of conditions	0.29	0.28	0.30
Two types of conditions	0.13	0.12	0.14
Three or more types of conditions	0.08	0.08	0.09
Number of observations	24,520		

Population estimates for England and Wales. Standard errors adjusted for clustering and stratification.

^1^The nine types of conditions are: respiratory, cancers, arthritis, obesity related, cardiovascular, other mental health conditions, psychiatric conditions, autoimmunity, and other chronic health condition.

Source: Understanding Society (2020), Wave 10, linked with ONSPD (Nov 2020) and various indicators of neighbourhood disadvantage in 2019 at the 2011 LSOA level.

We find that a similar population share is affected by multimorbidity when we use an alternative classification where the 35 health conditions are first grouped into types. According to this classification, 79% of the population are not classified as multimorbid. 13% have been diagnosed with two, and 8% with three or more of the nine types of conditions. We do not observe substantial differences in the socio-demographic characteristics of those defined as multimorbid on these different measures. Henceforward, we will focus our empirical analysis on the more straightforward count measure.

Table 3 reports the population share with multimorbidity overall and split by physical and mental health conditions mix, by deciles of neighbourhood deprivation.

**Table 3:** Multimorbidity by deciles of Index of Multiple Deprivation (IMD 2019).

Deciles	Multimorbidity (> 1 conditions)			Multimorbidity by physical/mental conditions mix								
				Physical conditions only			Mental conditions only			Mixed		
	Mean	95%	CI	Mean	95%	CI	Mean	95%	CI	Mean	95%	CI
1st (most deprived)	0.29	0.26	0.32	0.22	0.19	0.25	0.02	0.01	0.03	0.05	0.04	0.07
2nd	0.27	0.24	0.30	0.20	0.18	0.23	0.01	0.01	0.02	0.05	0.04	0.06
3rd	0.26	0.23	0.29	0.18	0.16	0.21	0.02	0.01	0.02	0.06	0.05	0.08
4th	0.24	0.22	0.26	0.19	0.17	0.21	0.01	0.00	0.01	0.04	0.03	0.05
5th	0.26	0.23	0.28	0.18	0.16	0.20	0.02	0.01	0.02	0.06	0.04	0.07
6th	0.24	0.22	0.27	0.18	0.16	0.20	0.02	0.01	0.03	0.04	0.03	0.05
7th	0.25	0.22	0.27	0.19	0.17	0.21	0.02	0.01	0.03	0.04	0.03	0.05
8th	0.22	0.20	0.24	0.17	0.15	0.19	0.01	0.01	0.02	0.04	0.03	0.05
9th	0.22	0.20	0.24	0.17	0.15	0.19	0.01	0.01	0.01	0.04	0.03	0.06
10th (least deprived)	0.21	0.19	0.23	0.16	0.14	0.18	0.01	0.01	0.02	0.04	0.03	0.04
Total	0.24	0.24	0.25	0.18	0.18	0.19	0.01	0.01	0.02	0.05	0.04	0.05

Population estimates for England and Wales. Standard errors adjusted for clustering and stratification. Number of observations in each model: 24,520.

Source: Understanding Society (2020), Wave 10, linked with ONSPD (Nov 2020) and Index of Multiple Deprivation (2019) at the LSOA 2011 level.

The prevalence of multimorbidity is higher only in the most deprived (bottom) decile of the neighbourhood deprivation distribution compared to the least deprived (top) decile of the neighbourhood deprivation distribution. Multimorbidity of only physical conditions is considerably more prevalent in the 10% most deprived neighbourhoods than in the 30% least deprived neighbourhoods. There is no association between neighbourhood deprivation and multimorbidity of only mental conditions or a mix of physical and mental conditions.

**Figure 1:**
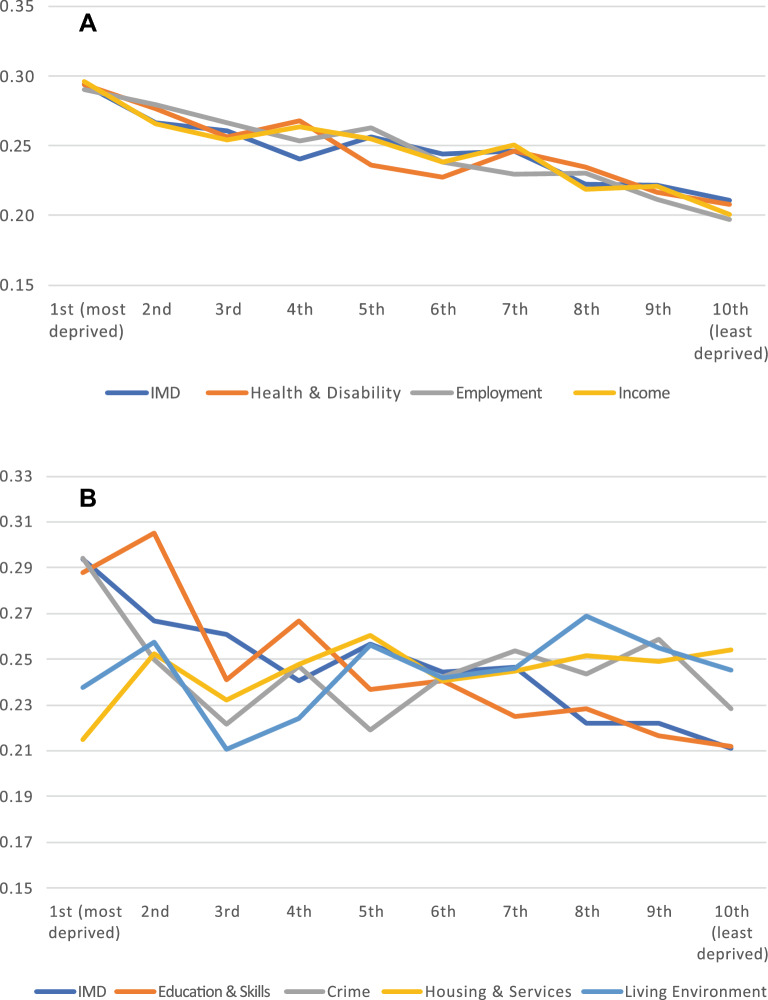
Multimorbidity rates by decile of neighbourhood deprivation domains. Panel (**A**) Health & disability, Employment, and Income domain. Panel (**B**) Education, Crime, Housing & Services, and Living Environment domain. Notes: Population estimates for England and Wales. Number of observations in each model: 24,520. For detailed results, see Supplementary Table S2 online. Source: Understanding Society (2020), Wave 10, linked with ONSPD (Nov 2020) and Index of Multiple Deprivation (2019) at the LSOA 2011 level.

Figure 1 shows the multimorbidity rates for each decile of the seven neighbourhood deprivation domains. Panel A presents the domains that we suspected might be closely linked to IMD and multimorbidity due to definitional overlap. Panel B presents the respective figures for domains where we did not suspect a close link.

The results suggest that the domains in Panel A closely track IMD rates across deciles, particularly at the bottom 30% and top 20% of the distribution. The distribution of the income domain resembles that of the IMD the most. By contrast, none of the domains in Panel B track IMD rates across deciles.

### Socioeconomic and demographic correlates of multimorbidity and multivariate regressions of multimorbidity on neighbourhood deprivation

Multimorbidity also varies by population characteristics (for detailed results, see Supplementary Table S2 online). While older people have higher multimorbidity rates in general, rates of multimorbidity involving only mental health conditions are more prevalent among younger age cohorts (that is, 2–3% compared to < 1% in older cohorts). The rates of multimorbidity involving only physical health conditions are the same as those for mental health only among age cohorts younger than 40 (that is, 2–5%).

Multimorbidity rates are highest among the White British population compared to their ethnic minority counterparts. Although there is not generally an association between multimorbidity and the social class of the current occupation, rates of multimorbidity are clearly higher among those who are out of the labour force—be it that they are retired (43%) or that they have longstanding health condition or disability (65%). Moreover, the long-term sick and disabled have higher rates of multimorbidity involving only mental health conditions (7%).

Finally, there are some associations between multimorbidity and place, that is, the retail and occupational environment in which participants live. We find that multimorbidity rates are particularly high among those living in busy urban retail centres, compared to those living in cosmopolitan metro-suburban mixed areas and those living in independent professional metropolitan service areas. Those living in retail areas also have elevated rates of mixed multimorbidity compared to those living in primarily residential suburban areas, ‘servants of society’-areas, and those living in cosmopolitan metro-suburban mixed areas.

Next, we performed logistic regressions of two multimorbidity measures on neighbourhood deprivation overall and domains of neighbourhood deprivation. The first multimorbidity measure, presented in the top half of Table 4, considers only physical conditions. In contrast, the second measure considers both physical and mental conditions (presented in the bottom half of Table 4). As coefficients from logistic regressions do not lend themselves to straightforward interpretation, we report relative marginal effects. Relative marginal effects (ME) express by how many percentage points the average probability would change if the explanatory characteristic changes by a unit, holding all else constant. For categorical variables, MEs express how much the probability would change if we were to observe a discrete change away from the base category. The baseline predicted probability of multimorbidity, calculated at the mean of the explanatory variables, and the population means provide a reference point for whether or not the MEs are small or large.

**Table 4:** Logistic regressions of multimorbidity on deciles of neighbourhood deprivation (IMD 2019 and domains). Marginal Effects (ME).

Multimorbidity (physical conditions only)	Unadjusted				Adjusted				Mean
	Pr(y) = 1	ME	t-stat	N	Pr(y) = 1	ME	t-stat	N	
IMD	0.208	***−0.007***	(−5.25)	24,520	0.157	***−0.011***	(−8.94)	24,520	5.70
Health and disability domain	0.208	***−0.008***	(−5.88)	24,520	0.157	***−0.011***	(−8.46)	24,520	5.67
Employment domain	0.208	***−0.009***	(−6.86)	24,520	0.156	***−0.013***	(−9.92)	24,520	5.59
Income domain	0.208	***−0.008***	(−5.85)	24,520	0.156	***−0.013***	(−10.02)	24,520	5.66
Education domain	0.208	***−0.009***	(−6.27)	24,520	0.156	***−0.012***	(−9.73)	24,520	5.59
Crime domain	0.209	−0.001	(−0.97)	24,520	0.158	***−0.006***	(−4.39)	24,520	5.77
Barriers to housing & services domain	0.209	0.002	(1.24)	24,520	0.158	−0.000	(−0.29)	24,520	5.63
Living environment domain	0.209	*0.003*	(2.45)	24,520	0.158	0.001	(0.99)	24,520	5.67

Multimorbidity (physical and mental conditions)	Unadjusted				Adjusted				Mean
	Pr(y) = 1	ME	t-stat	N	Pr(y) = 1	ME	t-stat	N	
IMD	0.244	***−0.008***	(−5.26)	24,520	0.211	***−0.012***	(−7.90)	24,520	5.70
Health and disability domain	0.244	***−0.008***	(−5.87)	24,520	0.211	***−0.011***	(−7.35)	24,520	5.67
Employment domain	0.244	***−0.010***	(−6.76)	24,520	0.211	***−0.013***	(−8.77)	24,520	5.59
Income domain	0.244	***−0.008***	(−5.80)	24,520	0.211	***−0.014***	(−8.91)	24,520	5.66
Education domain	0.244	***−0.009***	(−6.12)	24,520	0.211	***−0.012***	(−8.50)	24,520	5.59
Crime domain	0.245	−0.002	(−1.37)	24,520	0.212	***−0.006***	(−4.06)	24,520	5.77
Barriers to housing & services domain	0.245	0.002	(1.75)	24,520	0.212	0.000	(0.15)	24,520	5.63
Living environment domain	0.245	0.002	(1.71)	24,520	0.212	0.000	(0.30)	24,520	5.67

Population estimates for England and Wales. Standard errors adjusted for clustering and stratification. All estimates that reach statistical significance (*p* < 0.05) in italics; estimates in bold are statistically significant at *p* < 0.001.

Source: Understanding Society (2020), Wave 10, linked with ONSPD (Nov 2020) and Index for Multiple Deprivation 2019 at the LSOA 2011 level.

The models predict a multimorbidity rate of 21% when only physical conditions are considered and when estimates are not adjusted for socioeconomic and demographic factors; the respective figure is 16% in adjusted models. Other things being equal, a move from one decile of neighbourhood deprivation to the next decile reduces the probability to be multimorbid by 0.007 percentage points. Effect strengths for the health, income and employment domains and the education domain are in the same ballpark and of equal statistical significance. Effect sizes in models adjusting for individual characteristics are slightly larger, albeit this is only revealed at a precision of 3 decimal points. Interestingly, the effect of crime reaches statistical significance in these models.

Shifting the focus to the predictions of multimorbidity when physical and mental conditions are considered, the models predict a multimorbidity rate of 24% in the unadjusted and 21% in adjusted models. The patterns in effect sizes match those observed for the physical-conditions-only multimorbidity measure. Overall, the precision of the estimates is somewhat reduced, owing to the overall greater socioeconomic and demographic heterogeneity in the multimorbid population suffering from mental conditions.

### Examining the extent of unreported or undiagnosed mental health conditions and their association with neighbourhood deprivation

Participants interviewed online reported a larger number of mental conditions on average than those interviewed face-to-face, controlling for age (results not reported). We do not find any difference in the reporting rates of chronic physical conditions, indicating that there may be an issue with under-reporting or undiagnosed mental conditions.

Analyses were repeated using a common mental disorder assessment collected in self-completion from all participants to examine whether there is an association between not reporting any diagnosed mental health issues and psychological distress caseness assessed by the GHQ. We report the results in Table 5.

**Table 5:** Regressions examining the associations between not reporting diagnosed health conditions and having a high GHQ-12 score or persistently high GHQ-12 score.

Panel A: Linear regression of the number of chronic health conditions on reporting a high GHQ-12												
	High GHQ-12 score						Persistently high GHQ-12 score					
	Any conditions		Physical conditions		Mental conditions		Any conditions		Physical conditions		Mental conditions	
	b-coef	SE	b-coef	SE	b-coef	SE	b-coef	SE	b-coef	SE	b-coef	SE
Age	**0.03**	(41.70)	**0.03**	(46.97)	**−0.00**	(−4.98)	**0.03**	(41.50)	**0.03**	(46.65)	**−0.00**	(−4.03)
High GHQ-12 score	**0.69**	(19.13)	**0.42**	(14.28)	**0.28**	(14.52)						
Persistently high GQH-12 score							**0.74**	(18.35)	**0.45**	(13.94)	**0.29**	(14.03)
Number of observations	23,385		23,385		23,385		21,160		21,160		21,160	
R-squared	0.153		0.182		0.050		0.159		0.187		0.053	

Panel B: Logistic regressions of decile of IMD on not having reported any diagnosed health conditions / no diagnosed physical health conditions / no diagnosed mental health conditions but having a high or persistently high GQH-12 score. Marginal effects												
	High GHQ-12 score						Persistently high GHQ-12 score					
	No conditions		No physical conditions		No mental conditions		No conditions		No physical conditions		No mental conditions	
	ME	SE	ME	SE	ME	SE	ME	SE	ME	SE	ME	SE
Age	***−0.00***	(−19.94)	***−0.00***	(−21.80)	***−0.00***	(−8.75)	***−0.00***	(−11.41)	***−0.00***	(−13.20)	***−0.00***	(-5.05)
IMD decile	0.00	(0.04)	−0.00	(−0.33)	***−0.01***	(−4.49)	−0.00	(−1.67)	−0.00	(−1.86)	***−0.00***	(-4.88)
Number of observations	23.385		23.385		23.385		21.160		21.160		21.160	
- with high GHQ score	4.571		4.571		4.571		3.911		3.911		3.911	
- with outcome = 1	1.937		2.200		3.776		725		861		1.529	

Notes: Population estimates for England and Wales. Standard errors (SE) adjusted for clustering and stratification. All estimates that reach statistical significance (*p* < 0.05) in italics; estimates in bold are statistically significant at *p* < 0.001.Models analysing persistently high GHQ-12 score use information collected in Wave 9, and longitudinal weights are applied to account for unequal repeat response probabilities.

Source: Understanding Society (2020), Wave 10, linked with ONSPD (Nov 2020) and Index for Multiple Deprivation 2019 at the LSOA 2011 level.

We find that having a high GHQ score is associated with having more physical and mental health conditions on average. Moreover, there is an association between not reporting any mental conditions and having a high GHQ score, controlling for age. There is no association between not reporting any chronic physical conditions and having a high GHQ score.

We also find that there is an association between not reporting mental conditions and living in more deprived neighbourhoods. The panel nature of the survey data allowed us to confirm that these results hold when we examine a subgroup of the sample who had a high GHQ score in the last year and in the current year. This group may be considered more likely to have a chronic mental health issue that may not yet have been medically diagnosed.

## Discussion

We confirm that multimorbidity, defined as the presence of more than one long term condition, is associated with the Index of Multiple Deprivation in a large representative study of England and Wales. However, while the strong association of multimorbidity with area-level deprivation has been recognised^23–25^, other aspects of our findings are new or less well described. Firstly, the associations were restricted to multimorbidity defined by long-term physical conditions and not multimorbidity that includes long-term mental conditions. Secondly, associations were limited to the health, income, education and employment domains of IMD, potentially suggesting that these associations may reflect some overlap in the definition.

While we confirm previous associations of physical multimorbidity with area level deprivation, the mental health multimorbidity findings are unexpected: firstly, we observe lower levels of mental health issues than we might have expected, and secondly, several studies suggest that we should expect to see an association with deprivation (e.g., ^11,23^). Most of the earlier studies use hospital or GP records while we are using self-report. It may be that we genuinely observe fewer people who have been diagnosed with mental health problems in the general population than in the population receiving medical treatment. The study design enabled an exploration of whether the number of reported mental and physical health conditions reported varied by whether the interview took place face-to-face or online and suggested that those interviewed online reported a larger number of mental but not physical health issues. However, the online sample may have varied in other important ways, such as being younger or living in a community with access to higher speed internet access. These data support the notion that there is some underreporting, specifically of mental health issues.

This is consistent with previous research on mental illness stigma^26^ and social desirability bias^27^: Participants try to avoid embarrassment and repercussions from disclosing sensitive information to the interviewer^28^. Non-reporting of mental conditions may help explain why we find a relatively low prevalence of diagnosed mental conditions in our general population sample. It could also underpin the lack of an association between mental health and area deprivation: Concerns to reveal sensitive information about mental health conditions may be particularly marked among socioeconomically advantaged individuals who tend to hold more negative views about mental illness^29,30^. By contrast, non-accessibility of mental health services and under-diagnosing may play a role in the more deprived neighbourhoods^31^.

Further, results indicate that those who live in deprived neighbourhoods may have a de facto lower likelihood to be diagnosed with a mental health issue, e.g., due to the lack of mental health provision. Our data do not allow us to throw further light on this.

The strongest associations of multimorbidity and IMD occur with the health, education and income domains. The association with health risks a tautology. In the past studies, removing the health domain had little effect on either the assignment of areas into their deprivation quintile or the relationship between area-based deprivation and health^12^. Here we see that associations occur independently of the health domain.

Associations of multimorbidity with education and income domain support the notion that deprivation is associated with this measure of health. However, mechanisms are unclear. While the prevalence of multimorbidity increases in age and older age groups achieve lower educational attainment levels in England and Wales, our observations are unlikely to simply be due to age as analyses were adjusted for age group. The association with the income and employment domain may reflect reverse causation, i.e., that mechanisms may relate to earlier retirement or reduced labour market attachment in those with poor health^16^ resulting in apparent associations between multimorbidity and residing in deprived areas.

The employment domain of index of multiple deprivation measures the proportion of the working age population in an area involuntarily excluded from the labour market and this may include sickness. Importantly, multimorbidity may invoke caring responsibility that result in those doing this caring contributing to the proportion out of the labour market. Additional mechanisms by which multimorbidity may be associated with income and employment domains include an inability of those with poor health to move away from deprived areas. Further there may be a mismatch of jobs and individuals and a lack of quality public services^32–34^. Cross-sectional analyses cannot describe the temporal nature of these associations, and longitudinal analyses are better placed to examine the impact of retirement due to health and impact on income and or residential mobility^35^.

Advantages of these analyses include collecting many long-term conditions that adhere to the definition of chronic conditions^36^ in a large population study with detailed information from adults of all ages. However, there are a number of potential limitations. We are limited to self-report of long-term conditions as we do not have data linked to health services. However, the collection of sub-clinical measures of common mental disorder can be considered in our analyses as they enabled us to examine the distribution of common mental disorder and thus provides an insight into processes not captured by health services use. We did not examine conditions such as pain, which is common^23^ but may be captured in conditions such as arthritis^34^. The survey may have missed common mental health issues, which are known to be linked to area deprivation such as alcohol dependence and drug misuse^11^, despite an option of ‘other’ mental health issue in the questionnaire. On the other hand, these conditions often go undiagnosed and untreated^37^, hence would be altogether missed in health service use records. Overall, mental health multimorbidity had low prevalence (1% in the population; N = 345 cases in the sample), and we may not have had enough power to see associations between this type of multimorbidity and IMD decile. However, it suggests that currently there may be a lower public health burden in comparison to physical multimorbidity. We know that this is changing as there is a literature on the increasing prevalence of poor mental health in young people^38^, and there is a suggestion of more multimorbidity in younger age groups in our data. This may further increase in time as evidence suggests that diagnosis of a second mental health condition following an initial diagnosis is lagged, for example a recent study reported that in those diagnosed with a mood disorder under aged 20, were at nearly 40% risk for diagnosis of a neurotic condition within 5 years^39^.

In conclusion we find that physical multimorbidity represents a substantial population burden, particularly in the most deprived areas in England and Wales. Our findings suggest that mental health multimorbidity, which may be undiagnosed, may become a public health priority in the future.

## Supplementary Information

Supplementary Information.

## Data Availability

All data used in this paper are available for secondary data analysis (subject to access restrictions). The terms of the license prevent us from sharing the data directly with members of the public or other researchers. The large set of variables included in the data, the population sample frame and the postcodes of the addresses create a high risk of disclosing confidential information about individual respondents. To replicate our linked data set structure, analysts require secure access to the individual panel data and seek permission to access the exact postcodes of the sample members' addresses in their application. Information on how to access the *Understanding Society* panel data is provided on the study homepage; see https://www.understandingsociety.ac.uk/documentation/access-data. They also need to prepare a postcode-level data file containing the neighbourhood data and ask for the data to be uploaded to the secure server. To prepare the postcode-level file, analysts require access to the Office for National Statistics (ONS) Postcode Directory to extract the LSOA 2011 and Workplace Zone identifiers. The Index of Multiple Deprivation 2019 (at the LSOA 2011 level) is available for download from the Consumer Data Research Centre (https://www.cdrc.ac.uk/). To download the ONS Classification of Workplace Zones 2011 for England and Wales, visit http://cowz.geodata.soton.ac.uk/.
