# Supplementary material for: Multimorbidity is associated with the income, education, employment and health domains of area-level deprivation in adult residents in the UK
Source: Sci Rep. 2022 May 4;12:7280. doi: 10.1038/s41598-022-11310-9 (PMC9068903; doi:10.1038/s41598-022-11310-9)
Supplement: Supplementary file 1 — Supplementary Information. [file 41598_2022_11310_MOESM1_ESM.docx]

**Online Supplement**

**Supplementary Table S1: Descriptive sample statistics**

| Variable | Description | Mean | SD | Min | Max | N |
| --- | --- | --- | --- | --- | --- | --- |
| Age | Derived from the date of birth and interview date. (age_dv) | 51 | 18 | 16 | 103 | 24,520 |
| … by age group |  |  |  |  |  |  |
| *16-29 years* |  | 0.15 | 0.36 | 0 | 1 | 4,500 |
| *30-49 years* |  | 0.32 | 0.46 | 0 | 1 | 8,505 |
| *50-69 years* |  | 0.35 | 0.48 | 0 | 1 | 7,735 |
| *70 years or older* |  | 0.18 | 0.39 | 0 | 1 | 3,780 |
| Female | Longitudinally edited information from the survey data base (sex_dv) | 0.56 | 0.50 | 0 | 1 | 13,731 |
| Male |  | 0.44 | 0.50 | 0 | 1 | 10,789 |
| Ethnic group | Recode of the variable ethnicity_dv |  |  |  |  |  |
| White British |  | 0.75 | 0.43 | 0 | 1 | 18,377 |
| Caribbean/African Black |  | 0.05 | 0.21 | 0 | 1 | 1,137 |
| Indian |  | 0.05 | 0.21 | 0 | 1 | 1,187 |
| Pakistani |  | 0.04 | 0.21 | 0 | 1 | 1,090 |
| Bangladeshi |  | 0.02 | 0.15 | 0 | 1 | 582 |
| Other Asian |  | 0.02 | 0.12 | 0 | 1 | 370 |
| Other |  | 0.07 | 0.26 | 0 | 1 | 1,777 |
| *Continues next page* | | | | | | |

**Supplementary Table S1: (continued)**

| Variable | Description | Mean | SD | Min | Max | N |
| --- | --- | --- | --- | --- | --- | --- |
| Socio-economic classification | Recode of the variable nssec8_dv. Those not currently employed (i.e., nnsec8_dv= -8) are further categorised as [9] retired, [10] long-term sick or disabled, or [11] Other not currently employed, based on the participant’s reported main economic status (jbstat). |  |  |  |  |  |
| Large employers & higher management |  | 0.03 | 0.16 | 0 | 1 | 634 |
| Higher professional |  | 0.05 | 0.22 | 0 | 1 | 1,260 |
| Lower management & professional |  | 0.17 | 0.38 | 0 | 1 | 4,208 |
| Intermediate |  | 0.08 | 0.27 | 0 | 1 | 1,884 |
| Small employers & own account |  | 0.06 | 0.23 | 0 | 1 | 1,398 |
| Lower supervisory & technical |  | 0.03 | 0.18 | 0 | 1 | 837 |
| Semi-routine |  | 0.09 | 0.29 | 0 | 1 | 2,285 |
| Routine |  | 0.05 | 0.21 | 0 | 1 | 1,156 |
| Retired |  | 0.26 | 0.44 | 0 | 1 | 6,407 |
| Long-term sick / disabled |  | 0.03 | 0.17 | 0 | 1 | 766 |
| Other not currently employed |  | 0.15 | 0.36 | 0 | 1 | 3,685 |
| *Continues next page* | | | | | | |

**Supplementary Table S1: (continued)**

| Variable | Description | Mean | SD | Min | Max | N |
| --- | --- | --- | --- | --- | --- | --- |
| Classification of Workplace zones | Linked to Understanding Society using the Workplace Zone code in the ONSPD Nov 2020. Group and supergroup descriptions taken from [^34^](#_ENREF_34) |  |  |  |  |  |
| 1 Retail | Characterised by high workplace population density, higher than average female, young and student workers, working part-time, in semi-routine occupations in the retail, motor repair, real estate, food and accommodation services, and finance and insurance sectors. They tend to travel very short distances to work by public transport, bike or on foot. | 0.02 | 0.15 | 0 | 1 | 534 |
| 2 Top jobs | Characterised by high density and a low OA: WZ ratio (splits), higher than average percentage of young females, high Black, Asian + European ethnicities, high percentages of Level 4 qualifications, high employees, high on ICT, Finance, Prof/Sci/Tech, high on Higher managerial and Lower managerial, low on routine occupations, high on travelling more than 20km to work, low on working from home/no fixed place, low on part time working. This group is mainly found in large numbers in the major metropolitan centres. There are small numbers of isolated WZs in this group in other locations e.g. Science Parks etc. | 0.01 | 0.11 | 0 | 1 | 321 |
| 3a Metro suburban distribution | Above average Black and Asian ethnicities and post-2001 EU accession countries in a workforce which is average in very many dimensions but in which transport and storage is the most prominent industry, wholesale and retail, accommodation and food services are also above average. Scattered across outer suburban areas of major metropolitan centres. | 0.06 | 0.24 | 0 | 1 | 1,485 |
| *Continues next page* | | | | | | |

**Supplementary Table S1: (continued)**

| Variable | Description | Mean | SD | Min | Max | N |
| --- | --- | --- | --- | --- | --- | --- |
| 3b Cosmopolitan metro suburban mix | Above average Black and Asian ethnicities and post-2001 EU accession countries in a workforce in which construction, transport and storage, ICT are all above average as is self-employment without employees. Strongly marked by working from home or at no fixed place. Occurring in quite dense bands around the outer edges of multicultural urban areas and forming distinctive concentric rings around cities such as London, Birmingham and Leicester. | 0.11 | 0.31 | 0 | 1 | 2,725 |
| 3c Independent professional metro services | A multicultural workforce in which Black, Asian and European groups are all above average, as is self-employment. ICT, financial and insurance activities and professional services are all above the average, as are higher status occupations. Working from home or no fixed place and travel by public transport are all high. A very concentrated geographical distribution limited to especially inner suburban areas of London and a few major cities but entirely absent elsewhere. | 0.04 | 0.20 | 0 | 1 | 1,049 |
| 3d Suburban metro infrastructure | A multicultural workforce with above average levels of employment in education, health and social work activities and with lower managerial, administrative and technical occupations slightly above average. Widely dispersed in the suburban areas of major multicultural cities but entirely absent elsewhere. | 0.07 | 0.26 | 0 | 1 | 1,826 |
| 4a Non-metropolitan suburban areas | Mostly White suburban areas with employment across a range of industries but with local services such as education, health and public administration all above average. Above average female participation in the workforce, mainly lowest status occupations, low long-distance commuting and high percentages travelling under 5km, working from home or no fixed place. Widespread suburban distribution, but mostly away from the larger metropolitan centres. | 0.15 | 0.35 | 0 | 1 | 3,622 |
| *Continues next page* | | | | | | |

**Supplementary Table S1: (continued)**

| Variable | Description | Mean | SD | Min | Max | N |
| --- | --- | --- | --- | --- | --- | --- |
| 4b Primarily residential suburbs | Mostly white suburban areas with above average levels of self-employment without employees, ranging across many industries with energy, utilities, construction, ICT and education all above average. Very little commuting over 20km but very high levels of working from home or no fixed place. Widely dispersed geographical distribution, but mostly on the edges of larger urban areas. | 0.16 | 0.37 | 0 | 1 | 4,015 |
| 5 Manufacturing and distribution | The manufacturing, transport and distribution supergroup is characterised by a slightly lower than average OA:WZ ratio, low percentage of female workers, low percentage high qualifications, high percentage low qualifications, high employees, low part-time, high manufacturing, Energy/utilities, transport and storage, lower status working, low on working from home and no fixed place of work, slightly high on >20km. | 0.05 | 0.22 | 0 | 1 | 1,296 |
| 6a Rural with core services | Low female participation in a workforce which is older, mostly White and dominated by agriculture, forestry and fishing activities but where education and health are also above average, as is working from home. These are very widespread but tend to be found in rural service centres rather than in the most remote areas. | 0.05 | 0.21 | 0 | 1 | 1,185 |
| 6b Rural with non-local workers | Low female participation in a workforce which is older, mostly White and dominated by agriculture, forestry and fishing activities but with manufacturing and energy and utilities industries also present. Above average working from home. Widespread but not usually in the most remote rural areas. | 0.03 | 0.18 | 0 | 1 | 813 |
| 6c Rural with mining or quarrying | Low female participation in a workforce which is older, mostly White and dominated by agriculture, forestry and fishing activities but in combination with mining and quarrying activities as well as energy and construction. Above average working from home or no fixed place. Widespread and low density, often in remote areas. | 0.08 | 0.27 | 0 | 1 | 1,986 |
| *Continues next page* | | | | | | |

**Supplementary Table S1: (continued)**

| Variable | Description | Mean | SD | Min | Max | N |
| --- | --- | --- | --- | --- | --- | --- |
| 6d Traditional countryside | In many way the most extreme rural profile, with above average White, elderly workforce, self-employment and working from home. Industrial structure dominated by agriculture, forestry and fishing, with some energy and construction. Geographically extensive and widely dispersed, covering many of the most remote rural areas. | 0.10 | 0.30 | 0 | 1 | 2,373 |
| 7 Servants of society | The Servants of Society cluster is characterised by above average density, females and young females. It is just below average on non-White and European groups and just above for White. Level 4 qualifications are high, as are employees, while self-employment of any type is low. Both extremes of working hours are low, with part time 16-30 just on the average. The only industrial sectors above average are public administration and defence, education and health. Higher managerial jobs are a little above average, but lower managerial and intermediate status more so. Both extremes of travel distance are above average but working from home and without a fixed place of work are well below. In summary, these tend to be higher status, well-qualified employees in large public service organizations with good female representation in the workforce. They include hospitals, schools and colleges, prisons and government offices and are widely spread nationally, although somewhat clustered in the major service centres. | 0.05 | 0.22 | 0 | 1 | 1,290 |
| Number of chronic health conditions | Count of self-reported chronic health conditions | 0.94 | 1.34 | 0 | 13 | 24,520 |
| Number of physical conditions | Count of self-reported chronic physical health conditions | 0.82 | 1.19 | 0 | 13 | 24,520 |
| Number of mental conditions | Count of self-reported chronic mental health conditions | 0.12 | 0.49 | 0 | 6 | 24,520 |
| *Continues next page* | | | | | | |

**Supplementary Table S1: (continued)**

| Variable | Description | Mean | SD | Min | Max | N |
| --- | --- | --- | --- | --- | --- | --- |
| Multimorbidity (physical conditions only) | Indicator variable assuming a value of 1 if number of physical health conditions >1, else 0. | 0.20 | 0.40 | 0 | 1 | 24,520 |
| Multimorbidity (any conditions) | Indicator variable assuming a value of 1 if number of physical and mental health conditions >1, else 0. | 0.23 | 0.42 | 0 | 1 | 24,520 |
| Deciles of deprivation |  |  |  |  |  |  |
| Index of Multiple Deprivation (IMD) | Summary score of seven domains (weight in brackets): Income (22.5%), Employment (22.5%), Education and Skills (13.5%), Health and Disability (13.5%), Crime (9.3%), Barriers to Housing and Services (9.3%), Living environment (9.3%). | 5.50 | 2.88 | 1 | 10 | 24,520 |
| … by decile group |  |  |  |  |  |  |
| most deprived (deciles 1-3) |  | 0.31 | 0.46 | 0 | 1 | 7,501 |
| middling (deciles 4-7) |  | 0.39 | 0.49 | 0 | 1 | 9,599 |
| least deprived (deciles 8-10) |  | 0.30 | 0.46 | 0 | 1 | 7,420 |
| Income | Adults & children in Income Support families; Adults & children in Income-based Jobseeker 's Allowance families or Income-based Employment and Support Allowance families; Adults & children in Pension Credit (Guarantee) families; Adults & children in Child Tax Credit and Working Tax Credit families not already counted; Asylum seekers in England in receipt of subsistence support, accommodation support, or both; Adults & children in Universal Credit families where no adult is in 'Working - no requirements' conditionality regime. | 5.45 | 2.88 | 1 | 10 | 24,520 |
| … by decile group |  |  |  |  |  |  |
| most deprived (deciles 1-3) |  | 0.31 | 0.46 | 0 | 1 | 7,612 |
| middling (deciles 4-7) |  | 0.39 | 0.49 | 0 | 1 | 9,622 |
| least deprived (deciles 8-10) |  | 0.30 | 0.46 | 0 | 1 | 7,286 |
| *Continues next page* | | | | | | |

**Supplementary Table S1: (continued)**

| Variable | Description | Mean | SD | Min | Max | N |
| --- | --- | --- | --- | --- | --- | --- |
| Employment | Claimants of Jobseeker's Allowance; Claimants of Employment and Support Allowance; Claimants of Incapacity Benefit; Claimants of Severe Disablement Allowance; Claimants of Carer’s Allowance; Claimants of Universal Credit in the 'Searching for work' and 'No work requirements' conditionality groups. | 5.47 | 2.82 | 1 | 10 | 24,520 |
| … by decile group |  |  |  |  |  |  |
| most deprived (deciles 1-3) |  | 0.30 | 0.46 | 0 | 1 | 7,275 |
| middling (deciles 4-7) |  | 0.41 | 0.49 | 0 | 1 | 10,088 |
| least deprived (deciles 8-10) |  | 0.29 | 0.45 | 0 | 1 | 7,157 |
| Education | **Children & young people:** Key stage 2 attainment; Key stage 4 attainment; Secondary school absence; Staying on in education**;** Entry to higher education**; Adults skills:** Adults with no or low qualifications; English language proficiency. | 5.50 | 2.83 | 1 | 10 | 24,520 |
| … by decile group |  |  |  |  |  |  |
| most deprived (deciles 1-3) |  | 0.42 | 0.49 | 0 | 1 | 10,185 |
| middling (deciles 4-7) |  | 0.29 | 0.45 | 0 | 1 | 7,158 |
| least deprived (deciles 8-10) |  | 0.29 | 0.46 | 0 | 1 | 7,177 |
| Health deprivation and disability | Number of years of potential life lost; Comparative illness and disability ratio; Acute morbidity; Mood and anxiety disorders. Single score obtained from applying weights to each indicator following a factor analysis. | 5.59 | 2.81 | 1 | 10 | 24,520 |
| … by decile group |  |  |  |  |  |  |
| most deprived (deciles 1-3) |  | 0.28 | 0.45 | 0 | 1 | 6,900 |
| middling (deciles 4-7) |  | 0.42 | 0.49 | 0 | 1 | 10,226 |
| least deprived (deciles 8-10) |  | 0.30 | 0.46 | 0 | 1 | 7,394 |
| *Continues next page* | | | | | | |

**Supplementary Table S1: (continued)**

| Variable | Description | Mean | SD | Min | Max | N |
| --- | --- | --- | --- | --- | --- | --- |
| Crime | Recorded crime rates for: Violence Burglary, Theft, Criminal damage | 5.59 | 2.86 | 1 | 10 | 24,520 |
| … by decile group |  |  |  |  |  |  |
| most deprived (deciles 1-3) |  | 0.29 | 0.45 | 0 | 1 | 7,040 |
| middling (deciles 4-7) |  | 0.40 | 0.49 | 0 | 1 | 9,883 |
| least deprived (deciles 8-10) |  | 0.31 | 0.46 | 0 | 1 | 7,597 |
| Barriers to Housing and Services | **Geographical barriers:** Road distance to post office, primary school, general store or supermarket, GP surgery; **Wider barriers:** Household overcrowding; Homelessness; Housing affordability | 5.40 | 2.89 | 1 | 10 | 24,520 |
| … by decile group |  |  |  |  |  |  |
| most deprived (deciles 1-3) |  | 0.32 | 0.46 | 0 | 1 | 7,736 |
| middling (deciles 4-7) |  | 0.39 | 0.49 | 0 | 1 | 9,581 |
| least deprived (deciles 8-10) |  | 0.29 | 0.46 | 0 | 1 | 7,203 |
| Living environment | **Indoors living environment**: Housing in poor condition; Houses without central heating; **Outdoors living environment:** Air quality; Road traffic accidents. | 5.40 | 2.86 | 1 | 10 | 24,520 |
| … by decile group |  |  |  |  |  |  |
| most deprived (deciles 1-3) |  | 0.31 | 0.46 | 0 | 1 | 7,564 |
| middling (deciles 4-7) |  | 0.41 | 0.49 | 0 | 1 | 9,993 |
| least deprived (deciles 8-10) |  | 0.28 | 0.45 | 0 | 1 | 6,963 |
| Source: Understanding Society (2020), Wave 10, linked with ONSPD (Nov 2020), IMD 2019 and COWZ 2011 for England and Wales. | | | | | | |

**Supplementary Table S2: Mulitmorbidity by socio-demographic characteristics and domains of neighbourhood deprivation**

| Panel A: Health deprivation and Disability | | | | | | | | | | | | |
| --- | --- | --- | --- | --- | --- | --- | --- | --- | --- | --- | --- | --- |
| Deciles | Multimorbidity (>1 conditions) | | | Multimorbidity by physical / mental conditions mix | | | | | | | | |
|  |  |  |  | Physical conditions only | | | Mental conditions only | | | Mixed | | |
|  | % | 95% CI | | % | 95% CI | | % | 95% CI | | % | 95% CI | |
| 1^st^ (most deprived) | 0.29 | 0.26 | 0.32 | 0.22 | 0.19 | 0.24 | 0.02 | 0.01 | 0.03 | 0.06 | 0.04 | 0.07 |
| 2^nd^ | 0.28 | 0.25 | 0.31 | 0.21 | 0.19 | 0.24 | 0.01 | 0.01 | 0.02 | 0.06 | 0.04 | 0.07 |
| 3^rd^ | 0.27 | 0.24 | 0.29 | 0.20 | 0.18 | 0.22 | 0.02 | 0.01 | 0.03 | 0.05 | 0.04 | 0.06 |
| 4^th^ | 0.25 | 0.23 | 0.28 | 0.18 | 0.16 | 0.20 | 0.01 | 0.01 | 0.02 | 0.06 | 0.05 | 0.07 |
| 5^th^ | 0.26 | 0.24 | 0.29 | 0.20 | 0.18 | 0.22 | 0.01 | 0.01 | 0.02 | 0.05 | 0.04 | 0.07 |
| 6^th^ | 0.24 | 0.22 | 0.26 | 0.18 | 0.16 | 0.20 | 0.02 | 0.01 | 0.03 | 0.04 | 0.03 | 0.05 |
| 7^th^ | 0.23 | 0.21 | 0.25 | 0.17 | 0.15 | 0.19 | 0.02 | 0.01 | 0.02 | 0.04 | 0.03 | 0.05 |
| 8^th^ | 0.23 | 0.21 | 0.25 | 0.17 | 0.15 | 0.19 | 0.01 | 0.01 | 0.02 | 0.05 | 0.04 | 0.06 |
| 9^th^ | 0.21 | 0.19 | 0.23 | 0.16 | 0.14 | 0.18 | 0.01 | 0.01 | 0.02 | 0.04 | 0.03 | 0.05 |
| 10^th^ (least deprived) | 0.20 | 0.17 | 0.22 | 0.14 | 0.12 | 0.16 | 0.01 | 0.01 | 0.02 | 0.04 | 0.03 | 0.05 |
| *Continues next page* | | | | | | | | | | | | |

**Supplementary Table S2: Continued**

| Panel B: Employment | | | | | | | | | | | | |
| --- | --- | --- | --- | --- | --- | --- | --- | --- | --- | --- | --- | --- |
| Deciles | Multimorbidity (>1 conditions) | | | Multimorbidity by physical / mental conditions mix | | | | | | | | |
|  |  |  |  | Physical conditions only | | | Mental conditions only | | | Mixed | | |
|  | % | 95% CI | | % | 95% CI | | % | 95% CI | | % | 95% CI | |
| 1^st^ (most deprived) | 0.29 | 0.26 | 0.32 | 0.22 | 0.19 | 0.24 | 0.02 | 0.01 | 0.03 | 0.06 | 0.04 | 0.07 |
| 2^nd^ | 0.28 | 0.25 | 0.31 | 0.21 | 0.19 | 0.24 | 0.01 | 0.01 | 0.02 | 0.06 | 0.04 | 0.07 |
| 3^rd^ | 0.27 | 0.24 | 0.29 | 0.20 | 0.18 | 0.22 | 0.02 | 0.01 | 0.03 | 0.05 | 0.04 | 0.06 |
| 4^th^ | 0.25 | 0.23 | 0.28 | 0.18 | 0.16 | 0.20 | 0.01 | 0.01 | 0.02 | 0.06 | 0.05 | 0.07 |
| 5^th^ | 0.26 | 0.24 | 0.29 | 0.20 | 0.18 | 0.22 | 0.01 | 0.01 | 0.02 | 0.05 | 0.04 | 0.07 |
| 6^th^ | 0.24 | 0.22 | 0.26 | 0.18 | 0.16 | 0.20 | 0.02 | 0.01 | 0.03 | 0.04 | 0.03 | 0.05 |
| 7^th^ | 0.23 | 0.21 | 0.25 | 0.17 | 0.15 | 0.19 | 0.02 | 0.01 | 0.02 | 0.04 | 0.03 | 0.05 |
| 8^th^ | 0.23 | 0.21 | 0.25 | 0.17 | 0.15 | 0.19 | 0.01 | 0.01 | 0.02 | 0.05 | 0.04 | 0.06 |
| 9^th^ | 0.21 | 0.19 | 0.23 | 0.16 | 0.14 | 0.18 | 0.01 | 0.01 | 0.02 | 0.04 | 0.03 | 0.05 |
| 10^th^ (least deprived) | 0.20 | 0.17 | 0.22 | 0.14 | 0.12 | 0.16 | 0.01 | 0.01 | 0.02 | 0.04 | 0.03 | 0.05 |
| *Continues next page* | | | | | | | | | | | | |

**Supplementary Table S2: Continued**

| Panel C: Income | | | | | | | | | | | | |
| --- | --- | --- | --- | --- | --- | --- | --- | --- | --- | --- | --- | --- |
| Deciles | Multimorbidity (>1 conditions) | | | Multimorbidity by physical / mental conditions mix | | | | | | | | |
|  |  |  |  | Physical conditions only | | | Mental conditions only | | | Mixed | | |
|  | % | 95% CI | | % | 95% CI | | % | 95% CI | | % | 95% CI | |
| 1^st^ (most deprived) | 0.30 | 0.26 | 0.33 | 0.22 | 0.20 | 0.25 | 0.02 | 0.01 | 0.03 | 0.05 | 0.04 | 0.07 |
| 2^nd^ | 0.27 | 0.24 | 0.29 | 0.20 | 0.18 | 0.23 | 0.01 | 0.00 | 0.02 | 0.05 | 0.04 | 0.07 |
| 3^rd^ | 0.25 | 0.23 | 0.28 | 0.17 | 0.15 | 0.20 | 0.02 | 0.01 | 0.02 | 0.06 | 0.05 | 0.08 |
| 4^th^ | 0.26 | 0.24 | 0.29 | 0.20 | 0.18 | 0.22 | 0.01 | 0.01 | 0.02 | 0.05 | 0.04 | 0.06 |
| 5^th^ | 0.26 | 0.23 | 0.28 | 0.19 | 0.16 | 0.21 | 0.02 | 0.01 | 0.02 | 0.05 | 0.04 | 0.06 |
| 6^th^ | 0.24 | 0.22 | 0.26 | 0.18 | 0.16 | 0.20 | 0.02 | 0.01 | 0.02 | 0.05 | 0.03 | 0.06 |
| 7^th^ | 0.25 | 0.23 | 0.27 | 0.18 | 0.16 | 0.20 | 0.02 | 0.01 | 0.03 | 0.05 | 0.04 | 0.06 |
| 8^th^ | 0.22 | 0.20 | 0.24 | 0.17 | 0.16 | 0.19 | 0.01 | 0.00 | 0.01 | 0.04 | 0.03 | 0.05 |
| 9^th^ | 0.22 | 0.20 | 0.24 | 0.17 | 0.15 | 0.18 | 0.01 | 0.01 | 0.02 | 0.04 | 0.03 | 0.05 |
| 10^th^ (least deprived) | 0.20 | 0.18 | 0.22 | 0.15 | 0.13 | 0.17 | 0.01 | 0.01 | 0.02 | 0.04 | 0.03 | 0.05 |
| *Continues next page* | | | | | | | | | | | | |

**Supplementary Table S2: Continued**

| Panel D: Education | | | | | | | | | | | | |
| --- | --- | --- | --- | --- | --- | --- | --- | --- | --- | --- | --- | --- |
| Deciles | Multimorbidity (>1 conditions) | | | Multimorbidity by physical / mental conditions mix | | | | | | | | |
|  |  |  |  | Physical conditions only | | | Mental conditions only | | | Mixed | | |
|  | % | 95% CI | | % | 95% CI | | % | 95% CI | | % | 95% CI | |
| 1^st^ (most deprived) | 0.29 | 0.26 | 0.32 | 0.22 | 0.19 | 0.25 | 0.02 | 0.01 | 0.02 | 0.05 | 0.04 | 0.06 |
| 2^nd^ | 0.31 | 0.28 | 0.33 | 0.22 | 0.20 | 0.25 | 0.02 | 0.01 | 0.03 | 0.06 | 0.05 | 0.08 |
| 3^rd^ | 0.24 | 0.22 | 0.27 | 0.18 | 0.16 | 0.20 | 0.02 | 0.01 | 0.02 | 0.05 | 0.04 | 0.06 |
| 4^th^ | 0.27 | 0.24 | 0.29 | 0.21 | 0.19 | 0.23 | 0.01 | 0.01 | 0.02 | 0.05 | 0.03 | 0.06 |
| 5^th^ | 0.24 | 0.21 | 0.26 | 0.18 | 0.15 | 0.20 | 0.01 | 0.01 | 0.02 | 0.05 | 0.04 | 0.06 |
| 6^th^ | 0.24 | 0.22 | 0.26 | 0.17 | 0.16 | 0.19 | 0.02 | 0.01 | 0.02 | 0.05 | 0.04 | 0.06 |
| 7^th^ | 0.22 | 0.20 | 0.25 | 0.16 | 0.14 | 0.18 | 0.01 | 0.01 | 0.02 | 0.05 | 0.04 | 0.06 |
| 8^th^ | 0.23 | 0.21 | 0.25 | 0.17 | 0.16 | 0.19 | 0.01 | 0.00 | 0.02 | 0.04 | 0.03 | 0.06 |
| 9^th^ | 0.22 | 0.20 | 0.24 | 0.16 | 0.14 | 0.18 | 0.02 | 0.01 | 0.03 | 0.04 | 0.03 | 0.04 |
| 10^th^ (least deprived) | 0.21 | 0.19 | 0.23 | 0.16 | 0.14 | 0.18 | 0.02 | 0.01 | 0.02 | 0.04 | 0.03 | 0.05 |
| *Continues next page* | | | | | | | | | | | | |

**Supplementary Table S2: Continued**

| Panel E: Crime | | | | | | | | | | | | |
| --- | --- | --- | --- | --- | --- | --- | --- | --- | --- | --- | --- | --- |
| Deciles | Multimorbidity (>1 conditions) | | | Multimorbidity by physical / mental conditions mix | | | | | | | | |
|  |  |  |  | Physical conditions only | | | Mental conditions only | | | Mixed | | |
|  | % | 95% CI | | % | 95% CI | | % | 95% CI | | % | 95% CI | |
| 1^st^ (most deprived) | 0.29 | 0.26 | 0.32 | 0.22 | 0.19 | 0.24 | 0.02 | 0.01 | 0.03 | 0.06 | 0.04 | 0.07 |
| 2^nd^ | 0.25 | 0.22 | 0.28 | 0.19 | 0.16 | 0.21 | 0.02 | 0.01 | 0.02 | 0.05 | 0.04 | 0.06 |
| 3^rd^ | 0.22 | 0.20 | 0.25 | 0.17 | 0.15 | 0.19 | 0.01 | 0.01 | 0.02 | 0.04 | 0.03 | 0.05 |
| 4^th^ | 0.25 | 0.22 | 0.27 | 0.18 | 0.16 | 0.20 | 0.02 | 0.01 | 0.02 | 0.05 | 0.04 | 0.06 |
| 5^th^ | 0.22 | 0.20 | 0.24 | 0.16 | 0.14 | 0.18 | 0.01 | 0.01 | 0.02 | 0.04 | 0.03 | 0.05 |
| 6^th^ | 0.24 | 0.22 | 0.27 | 0.18 | 0.16 | 0.20 | 0.02 | 0.01 | 0.02 | 0.05 | 0.04 | 0.06 |
| 7^th^ | 0.25 | 0.23 | 0.28 | 0.18 | 0.16 | 0.20 | 0.02 | 0.01 | 0.02 | 0.05 | 0.04 | 0.07 |
| 8^th^ | 0.24 | 0.22 | 0.27 | 0.19 | 0.17 | 0.21 | 0.02 | 0.01 | 0.02 | 0.04 | 0.03 | 0.05 |
| 9^th^ | 0.26 | 0.24 | 0.28 | 0.19 | 0.17 | 0.21 | 0.01 | 0.01 | 0.02 | 0.05 | 0.04 | 0.06 |
| 10^th^ (least deprived) | 0.23 | 0.21 | 0.25 | 0.18 | 0.16 | 0.20 | 0.01 | 0.00 | 0.01 | 0.04 | 0.03 | 0.05 |
| *Continues next page* | | | | | | | | | | | | |

**Supplementary Table S2: Continued**

| Panel F: Barriers to Housing and Services | | | | | | | | | | | | |
| --- | --- | --- | --- | --- | --- | --- | --- | --- | --- | --- | --- | --- |
| Deciles | Multimorbidity (>1 conditions) | | | Multimorbidity by physical / mental conditions mix | | | | | | | | |
|  |  |  |  | Physical conditions only | | | Mental conditions only | | | Mixed | | |
|  | % | 95% CI | | % | 95% CI | | % | 95% CI | | % | 95% CI | |
| 1^st^ (most deprived) | 0.21 | 0.19 | 0.24 | 0.16 | 0.14 | 0.18 | 0.01 | 0.00 | 0.02 | 0.05 | 0.03 | 0.06 |
| 2^nd^ | 0.25 | 0.22 | 0.28 | 0.19 | 0.17 | 0.21 | 0.01 | 0.00 | 0.01 | 0.05 | 0.04 | 0.07 |
| 3^rd^ | 0.23 | 0.21 | 0.26 | 0.18 | 0.16 | 0.20 | 0.01 | 0.00 | 0.01 | 0.04 | 0.03 | 0.05 |
| 4^th^ | 0.25 | 0.22 | 0.27 | 0.19 | 0.17 | 0.21 | 0.01 | 0.01 | 0.02 | 0.05 | 0.04 | 0.06 |
| 5^th^ | 0.26 | 0.23 | 0.29 | 0.18 | 0.16 | 0.20 | 0.02 | 0.01 | 0.03 | 0.06 | 0.04 | 0.07 |
| 6^th^ | 0.24 | 0.22 | 0.26 | 0.18 | 0.16 | 0.20 | 0.02 | 0.01 | 0.03 | 0.04 | 0.03 | 0.05 |
| 7^th^ | 0.24 | 0.22 | 0.27 | 0.18 | 0.16 | 0.20 | 0.01 | 0.01 | 0.02 | 0.05 | 0.04 | 0.06 |
| 8^th^ | 0.25 | 0.23 | 0.28 | 0.19 | 0.17 | 0.21 | 0.02 | 0.01 | 0.03 | 0.04 | 0.03 | 0.05 |
| 9^th^ | 0.25 | 0.23 | 0.27 | 0.19 | 0.17 | 0.21 | 0.01 | 0.01 | 0.02 | 0.04 | 0.03 | 0.05 |
| 10^th^ (least deprived) | 0.25 | 0.23 | 0.28 | 0.18 | 0.16 | 0.20 | 0.02 | 0.01 | 0.02 | 0.06 | 0.04 | 0.07 |
| *Continues next page* | | | | | | | | | | | | |

**Supplementary Table S2: Continued**

| Panel G: Indoor and outdoor living environment | | | | | | | | | | | | |
| --- | --- | --- | --- | --- | --- | --- | --- | --- | --- | --- | --- | --- |
| Deciles | Multimorbidity (>1 conditions) | | | Multimorbidity by physical / mental conditions mix | | | | | | | | |
|  |  |  |  | Physical conditions only | | | Mental conditions only | | | Mixed | | |
|  | % | 95% CI | | % | 95% CI | | % | 95% CI | | % | 95% CI | |
| 1^st^ (most deprived) | 0.24 | 0.21 | 0.26 | 0.17 | 0.14 | 0.19 | 0.02 | 0.01 | 0.03 | 0.05 | 0.04 | 0.06 |
| 2^nd^ | 0.26 | 0.23 | 0.28 | 0.18 | 0.15 | 0.20 | 0.02 | 0.01 | 0.03 | 0.06 | 0.05 | 0.07 |
| 3^rd^ | 0.21 | 0.19 | 0.24 | 0.16 | 0.14 | 0.18 | 0.01 | 0.01 | 0.02 | 0.04 | 0.03 | 0.05 |
| 4^th^ | 0.22 | 0.20 | 0.25 | 0.17 | 0.15 | 0.20 | 0.01 | 0.01 | 0.02 | 0.04 | 0.03 | 0.05 |
| 5^th^ | 0.26 | 0.23 | 0.28 | 0.20 | 0.17 | 0.22 | 0.01 | 0.01 | 0.02 | 0.05 | 0.03 | 0.06 |
| 6^th^ | 0.24 | 0.22 | 0.27 | 0.18 | 0.16 | 0.21 | 0.01 | 0.01 | 0.02 | 0.04 | 0.03 | 0.05 |
| 7^th^ | 0.25 | 0.22 | 0.27 | 0.19 | 0.17 | 0.21 | 0.01 | 0.01 | 0.02 | 0.05 | 0.04 | 0.06 |
| 8^th^ | 0.27 | 0.25 | 0.29 | 0.21 | 0.18 | 0.23 | 0.01 | 0.01 | 0.02 | 0.05 | 0.04 | 0.06 |
| 9^th^ | 0.25 | 0.23 | 0.28 | 0.19 | 0.17 | 0.21 | 0.02 | 0.01 | 0.02 | 0.04 | 0.03 | 0.06 |
| 10^th^ (least deprived) | 0.25 | 0.22 | 0.27 | 0.18 | 0.16 | 0.20 | 0.01 | 0.00 | 0.01 | 0.05 | 0.04 | 0.07 |
| Total | 0.24 | 0.24 | 0.25 | 0.18 | 0.18 | 0.19 | 0.01 | 0.01 | 0.02 | 0.05 | 0.04 | 0.05 |
| *Continues next page* | | | | | | | | | | | | |

**Supplementary Table S2: Continued**

| Panel H: Multimorbidity by age group | | | | | | | | | | | | |
| --- | --- | --- | --- | --- | --- | --- | --- | --- | --- | --- | --- | --- |
| Age group | Multimorbidity (>1 conditions) | | | Multiple chronic health conditions | | | | | | | | |
|  |  |  |  | Physical conditions only | | | Mental conditions only | | | Mixed | | |
|  | % | 95% CI | | % | 95% CI | | % | 95% CI | | % | 95% CI | |
| 16-19 | 0.06 | 0.04 | 0.08 | 0.02 | 0.01 | 0.03 | 0.02 | 0.01 | 0.04 | 0.02 | 0.01 | 0.03 |
| 20-29 | 0.09 | 0.07 | 0.10 | 0.02 | 0.01 | 0.03 | 0.03 | 0.02 | 0.04 | 0.03 | 0.02 | 0.04 |
| 30-39 | 0.12 | 0.11 | 0.14 | 0.05 | 0.04 | 0.06 | 0.03 | 0.02 | 0.04 | 0.04 | 0.03 | 0.06 |
| 40-49 | 0.16 | 0.14 | 0.17 | 0.09 | 0.08 | 0.10 | 0.01 | 0.01 | 0.02 | 0.05 | 0.04 | 0.06 |
| 50-59 | 0.24 | 0.22 | 0.25 | 0.16 | 0.15 | 0.18 | 0.01 | 0.01 | 0.02 | 0.06 | 0.05 | 0.07 |
| 60-69 | 0.36 | 0.34 | 0.38 | 0.29 | 0.27 | 0.31 | 0.01 | 0.00 | 0.01 | 0.06 | 0.05 | 0.07 |
| 70-79 | 0.44 | 0.42 | 0.46 | 0.40 | 0.38 | 0.42 | 0.00 | 0.00 | 0.00 | 0.04 | 0.03 | 0.05 |
| 80 or older | 0.50 | 0.46 | 0.53 | 0.47 | 0.44 | 0.50 | 0.00 | 0.00 | 0.00 | 0.02 | 0.01 | 0.03 |
| Panel I: Multimorbidity by ethnicity group | | | | | | | | | | | | |
| Ethnic group | Multimorbidity (>1 conditions) | | | Multiple chronic health conditions | | | | | | | | |
|  |  |  |  | Physical conditions only | | | Mental conditions only | | | Mixed | | |
|  | % | 95% CI | | % | 95% CI | | % | 95% CI | | % | 95% CI | |
| White British | 0.25 | 0.25 | 0.26 | 0.19 | 0.18 | 0.20 | 0.02 | 0.01 | 0.02 | 0.05 | 0.05 | 0.05 |
| Caribbean/African Black | 0.17 | 0.13 | 0.20 | 0.13 | 0.10 | 0.17 | 0.01 | 0.00 | 0.01 | 0.03 | 0.01 | 0.04 |
| Indian | 0.13 | 0.10 | 0.16 | 0.11 | 0.08 | 0.14 | 0.00 | 0.00 | 0.01 | 0.02 | 0.01 | 0.03 |
| Pakistani | 0.11 | 0.08 | 0.14 | 0.09 | 0.06 | 0.11 | 0.00 | 0.00 | 0.01 | 0.02 | 0.01 | 0.03 |
| Bangladeshi | 0.09 | 0.05 | 0.13 | 0.07 | 0.04 | 0.11 | 0.01 | 0.00 | 0.01 | 0.01 | 0.00 | 0.02 |
| Other Asian | 0.10 | 0.06 | 0.13 | 0.08 | 0.05 | 0.11 | 0.00 | 0.00 | 0.01 | 0.02 | 0.00 | 0.03 |
| Other | 0.19 | 0.16 | 0.21 | 0.12 | 0.10 | 0.14 | 0.01 | 0.01 | 0.02 | 0.05 | 0.04 | 0.07 |
| *Continues next page* | | | | | | | | | | | | |

**Supplementary Table S2: Continued**

| Panel J: Multimorbidity by socio-economic classification of the current job | | | | | | | | | | | | |
| --- | --- | --- | --- | --- | --- | --- | --- | --- | --- | --- | --- | --- |
| Socio-economic group | Multimorbidity (>1 conditions) | | | Multiple chronic health conditions | | | | | | | | |
|  |  |  |  | Physical conditions only | | | Mental conditions only | | | Mixed | | |
|  | % | 95% CI | | % | 95% CI | | % | 95% CI | | % | 95% CI | |
| Large employers & higher management | 0.13 | 0.10 | 0.17 | 0.10 | 0.07 | 0.13 | 0.01 | -0.01 | 0.03 | 0.02 | 0.01 | 0.03 |
| Higher professional | 0.13 | 0.11 | 0.16 | 0.09 | 0.06 | 0.11 | 0.02 | 0.01 | 0.03 | 0.03 | 0.01 | 0.04 |
| Lower management & professional | 0.15 | 0.13 | 0.16 | 0.10 | 0.08 | 0.11 | 0.01 | 0.01 | 0.02 | 0.04 | 0.03 | 0.04 |
| Intermediate | 0.16 | 0.14 | 0.19 | 0.10 | 0.08 | 0.11 | 0.02 | 0.01 | 0.03 | 0.05 | 0.03 | 0.06 |
| Small employers & own account | 0.16 | 0.14 | 0.19 | 0.11 | 0.09 | 0.14 | 0.01 | 0.00 | 0.02 | 0.04 | 0.02 | 0.05 |
| Lower supervisory & technical | 0.13 | 0.10 | 0.17 | 0.09 | 0.07 | 0.12 | 0.02 | 0.00 | 0.03 | 0.02 | 0.01 | 0.04 |
| Semi-routine | 0.17 | 0.15 | 0.19 | 0.12 | 0.10 | 0.14 | 0.01 | 0.01 | 0.02 | 0.04 | 0.03 | 0.05 |
| Routine | 0.15 | 0.12 | 0.18 | 0.10 | 0.08 | 0.13 | 0.02 | 0.00 | 0.03 | 0.03 | 0.02 | 0.05 |
| Retired | 0.43 | 0.41 | 0.44 | 0.38 | 0.37 | 0.40 | 0.00 | 0.00 | 0.00 | 0.04 | 0.04 | 0.05 |
| Long-term sick / disabled | 0.65 | 0.60 | 0.70 | 0.29 | 0.24 | 0.33 | 0.07 | 0.04 | 0.10 | 0.29 | 0.24 | 0.33 |
| Other not currently employed | 0.15 | 0.13 | 0.17 | 0.08 | 0.07 | 0.10 | 0.02 | 0.02 | 0.03 | 0.04 | 0.03 | 0.05 |
| *Continues next page* | | | | | | | | | | | | |

**Supplementary Table S2: Continued**

| Panel K: Multimorbidity by area type | | | | | | | | | | | | |
| --- | --- | --- | --- | --- | --- | --- | --- | --- | --- | --- | --- | --- |
| Area type | Multimorbidity (>1 conditions) | | | Multiple chronic health conditions | | | | | | | | |
|  |  |  |  | Physical conditions only | | | Mental conditions only | | | Mixed | | |
|  | % | 95% CI | | % | 95% CI | | % | 95% CI | | % | 95% CI | |
| Retail | 0.31 | 0.25 | 0.36 | 0.19 | 0.14 | 0.23 | 0.03 | 0.01 | 0.05 | 0.09 | 0.06 | 0.12 |
| Top jobs | 0.20 | 0.14 | 0.27 | 0.10 | 0.05 | 0.14 | 0.03 | 0.01 | 0.05 | 0.08 | 0.02 | 0.13 |
| Metro suburban distribution | 0.22 | 0.17 | 0.26 | 0.15 | 0.12 | 0.19 | 0.01 | 0.00 | 0.03 | 0.05 | 0.03 | 0.07 |
| Cosmopolitan. metro suburban mix | 0.21 | 0.19 | 0.24 | 0.16 | 0.14 | 0.18 | 0.01 | 0.00 | 0.02 | 0.04 | 0.03 | 0.05 |
| Independent professional metro services | 0.20 | 0.17 | 0.24 | 0.15 | 0.12 | 0.18 | 0.01 | 0.00 | 0.01 | 0.05 | 0.03 | 0.07 |
| Suburban metro infrastructure | 0.26 | 0.22 | 0.29 | 0.20 | 0.17 | 0.24 | 0.01 | 0.00 | 0.02 | 0.04 | 0.03 | 0.06 |
| Non-metropolitan suburban areas | 0.26 | 0.24 | 0.28 | 0.20 | 0.18 | 0.21 | 0.02 | 0.01 | 0.02 | 0.05 | 0.04 | 0.06 |
| Primarily residential suburbs | 0.24 | 0.22 | 0.25 | 0.17 | 0.16 | 0.19 | 0.02 | 0.01 | 0.02 | 0.05 | 0.04 | 0.05 |
| Manufacturing and distribution | 0.23 | 0.20 | 0.26 | 0.17 | 0.15 | 0.20 | 0.01 | 0.00 | 0.02 | 0.04 | 0.03 | 0.06 |
| Rural with core services | 0.26 | 0.23 | 0.30 | 0.20 | 0.17 | 0.23 | 0.02 | 0.01 | 0.03 | 0.04 | 0.03 | 0.06 |
| Rural with non-local workers | 0.26 | 0.22 | 0.30 | 0.19 | 0.15 | 0.22 | 0.01 | 0.00 | 0.02 | 0.06 | 0.03 | 0.08 |
| Rural with mining or quarrying | 0.24 | 0.22 | 0.27 | 0.19 | 0.17 | 0.21 | 0.01 | 0.00 | 0.01 | 0.05 | 0.03 | 0.06 |
| Traditional countryside | 0.27 | 0.25 | 0.29 | 0.20 | 0.18 | 0.22 | 0.02 | 0.01 | 0.02 | 0.05 | 0.04 | 0.06 |
| Servants of society | 0.24 | 0.21 | 0.28 | 0.19 | 0.17 | 0.22 | 0.01 | 0.01 | 0.02 | 0.03 | 0.02 | 0.05 |
| Notes: Populations estimates for England and Wales. Standard errors adjusted for clustering and stratification. Number of observations: 24,520.  Source: Understanding Society (2020), Wave 10, linked with ONSPD (Nov 2020) and Index for Multiple Deprivation 2019 at the LSOA 2011 level. | | | | | | | | | | | | |
